# Caring for Children with Dravet Syndrome: Exploring the Daily Challenges of Family Caregivers

**DOI:** 10.3390/children10081410

**Published:** 2023-08-19

**Authors:** Jan Domaradzki, Dariusz Walkowiak

**Affiliations:** 1 Department of Social Sciences and Humanities, Poznan University of Medical Sciences, 60-806 Poznań, Poland; 2 Department of Organization and Management in Health Care, Poznan University of Medical Sciences, 60-356 Poznań, Poland; dariuszwalkowiak@ump.edu.pl

**Keywords:** caregiver burden, caregivers, children, Dravet syndrome, epilepsy, epilepsies, myoclonic

## Abstract

While Polish studies focus on the symptoms, causes and treatment of people suffering from Dravet syndrome (DS), much less is known about the situation of the family caregivers of DS children. This study was designed to explore the experiences, daily challenges and needs related to caring for DS children. An anonymous self-administered online questionnaire was developed. The survey was completed by 75 family caregivers affiliated with the Association for People with Severe Refractory Epilepsy DRAVET.PL on Facebook. Most caregivers felt burdened by their children’s reduced mobility (57.3%), mood swings (57.3%), lack of access to rehabilitation and medicine (56%) and healthcare expenses (50.7%). Caregivers also complained of a lack of time to themselves (76%) and work restrictions resulting from caregiving (72%). They consequently reported experiencing fatigue (84%), a deterioration of mental health (60%) and intimacy problems with their spouse/partner (53.4%). An important source of strain was a prolonged diagnostic odyssey and the constant struggle over the healthcare services for DS children. Since DS caregivers’ problems and needs are often overlooked, they may be described as the forgotten people in DS. Healthcare professionals should be educated about the challenges related to caring for DS child, psycho-social status and coping resources of DS caregivers, and should focus on identification, monitoring and supporting caregivers’ physical and mental well-being and needs.

## 1. Introduction

Dravet syndrome (DS) is a rare, severe and lifelong myoclonic epilepsy that usually begins in early infancy and affects 1 in 15–30,000 live births [1,2,3]. While it is one of the most common monogenic types of epilepsy, in about 80% of patients it is associated with a mutation in the SCN1A gene involved in neuronal signalling. At the same time, most such mutations occur de novo [4,5]. Because clinicians are cautious about making such a serious diagnosis before the disease manifests its clinical features, however, DS patients often experience delayed diagnosis, even as late as up to three years of age [6].

The main features of DS include multiple seizure types, even on a weekly or daily basis, prolonged, typically lateralised febrile and convulsive seizures that are often resistant to current anti-epileptic drugs, and frequent episodes of status epilepticus [2,6]. While initial seizures are often precipitated by fever and infections [2] other provoking factors include exposure to temperature changes (for example getting out of a bath), bright or flashing lights, warm weather, over-exertion and strong emotions [7,8]. Although seizures may decrease in late childhood and adulthood, since DS is highly pharmacotherapy-resistant and refractory, more than 90% of DS children do not achieve seizure freedom [9]. Thus, while DS shares some characteristics with other types of epilepsy, unlike many other diseases, the spectrum and type of seizures in DS are usually more frequent, prolonged, recurrent and more difficult to control [10].

While many children affected exhibit normal early development, seizures usually start within the first year of a child’s life. DS is also characterised by behavioural and sensory integration disorders, neuro-developmental delay and neurological disability that result from the seizures. Cognitive and motor system dysfunction also often persists into adulthood [2]. Other symptoms may include autism spectrum characteristics, ADHD, communication impairments, cardiovascular conditions, dysautonomia, cognitive dysfunction, disturbed sleep and motor impairment, or eating problems that often worsen during adolescence. Most teenagers and adults with DS are therefore dependent on caregivers [9,11,12,13,14].

DS treatment aims to reduce seizure frequency and prevent status epilepticus. While some antiepileptic medications for DS are available (i.e., sodium valproate, topiramate, stiripentol, fenfluramine, and cannabidiol), pharmacotherapy is often combined with ketogenic diet and vagus nerve stimulation [15,16].

DS children are at high risk of premature mortality due to fatal status epilepticus and accidents, making this a major concern for families and caregivers. The mortality rate in DS is 10% to 15% with age of death ranging from 3 to 27 [17]. Up to half of all deaths in DS patients also result from sudden unexpected death in epilepsy (SUDEP) that often occurs during sleep [2,17,18,19].

For all these reasons caring for a child with DS has wide-reaching psychosocial and economic consequences for family carers [11,13,14,20,21,22,23,24,25,26,27,28,29]. These problems, in turn, may affect caregivers’ ability to care for DS children. While Polish studies often focus on the symptoms, causes and treatment of DS patients; DS carers are often overlooked [30,31]. Thus, this study aims to explore the problems and needs of family caregivers of DS children in Poland.

## 2. Materials and Methods

### 2.1. Study Design

While previous research in Poland has focused on the clinical manifestation in DS patients, there remains a shortage of studies on the impact of caring for a DS child on family caregivers. Thus, this study was designed to give voice to Polish family caregivers and enable them to share their experiences.

After a thorough analysis of the literature an anonymous self-administered online questionnaire was developed to assess the challenges and needs related to caring for DS children. 

### 2.2. Participants and Setting

The survey was conducted between 30 December 2022 and 30 January 2023 among caregivers affiliated with the Association for People with Severe Refractory Epilepsy DRAVET.PL, a private support group for parents on Facebook.

Respondents were eligible to participate if they were aged over 18, were parents or family members of a child with DS (below 18 years of age) and provided care to a DS child, were willing to share their experiences as caregivers, were able to use electronic devices and complete the online questionnaire and provided the informed consent.

After consent was obtained, the survey was electronically administered to members of the Association for People with Severe Refractory Epilepsy DRAVET.PL on Facebook.

### 2.3. Research Tool

Since there is no special tool for assessing DS caregivers’ problems and needs, we have constructed an original questionnaire that was based on a review of the literature.

While the instrument used in this study was an ad hoc tool it was anchored in the guidelines of the European Statistical System [32]. At the same time, its design and administration were described in-depth elsewhere [33]. The questionnaire consisted of 19 open-ended questions designed to explore caregivers’ experiences, problems and needs related to providing care for a DS child. It was divided into several sections. The first posed questions about caregivers’ perception of problems related to raising a child with DS. The second section included questions regarding the social impact of DS. The third asked questions about caregivers’ encounters with the Polish healthcare system. The fourth part featured a series of demographic questions.

While questions were formulated in simple language, they used a 5-Likert scale, where 1 was strong disagreement or dissatisfaction and 5 strong agreement or satisfaction.

At the same time, while this research was part of a larger project focusing on the impact of caring for DS child on caregivers’ quality of life [33], the data presented here focuses on DS carers’ problems and needs, the social impact of DS and caregivers’ experiences with the healthcare system.

### 2.4. Data Collection

After permission to distribute a questionnaire was obtained from the board of the Association, the questionnaire was posted to its page on Facebook together with an invitation letter explaining the purpose and methods of the study, the anonymous and voluntary character of the survey, and the possibility to withdraw from the study at any time without any implications.

Participants’ responses were collected using a self-administered web questionnaire with the assistance of their mobile devices (e.g., smartphones or tablets). The survey took approximately 15 min to complete.

### 2.5. Statistical Methods

The data gathered from the survey were authenticated, reviewed for comprehensiveness, and then transferred into the statistical software JASP (Version 0.16.3). The findings are exhibited as descriptive statistics. To investigate the relationship between item scores, the Kendall rank correlation coefficient tau was employed as a hypothesis test. A 95% confidence interval (CI) for Kendall’s tau was determined through 10,000 bootstrap samples.

### 2.6. Ethical Issues

The survey was conducted in line with the principles of the Declaration of Helsinki. Ethics approval was given by Poznan University of Medical Sciences Bioethics Committee (KB–833/22, 22 October 2022). All eligible caregivers provided their informed consent.

## 3. Results

Because there is no single registry of DS children in Poland, nor does the Association for People with Severe Refractory Epilepsy DRAVET.PL possess such a registry, it was impossible to assess the exact number of respondents potentially eligible for the study. Despite this limitation, 75 family caregivers caring for 80 children with DS responded and completed the survey. Of these, 66 were mothers, 7 fathers and 2 were other relatives. (Table 1). The majority were aged between 30 and 49. Among the patients there were 37 female and 43 male children. The largest group of children was aged between 11 and 18 or 6 and 10 (38 and 28, respectively). While 80% of the caregivers used no extracurricular help for their DS child, 89.3% benefited from a caregivers’ allowance, which is granted to parents who need to give up work for a certain period of time while taking personal care of one’s family member who requires special care. A total of 86.7% of caregivers highlighted the severity of their DS children’s health condition and disability.

The reasons most often indicated by caregivers as challenging turned out to be reduced mobility (57.3%), the mood swings of the DS child (57.3%), a lack of access to rehabilitation (56%) and drug and healthcare expenses (50.7%) (Table 2). The most common causes of burnout were lack of time for oneself (76%), work restrictions (72%) and caregiving for DS child (58.7%). When asked about their problems, caregivers most often pointed to fatigue (84%), deterioration of mental health (60%) and intimacy problems with spouse/partner (53.4%).

**Figure 1:**
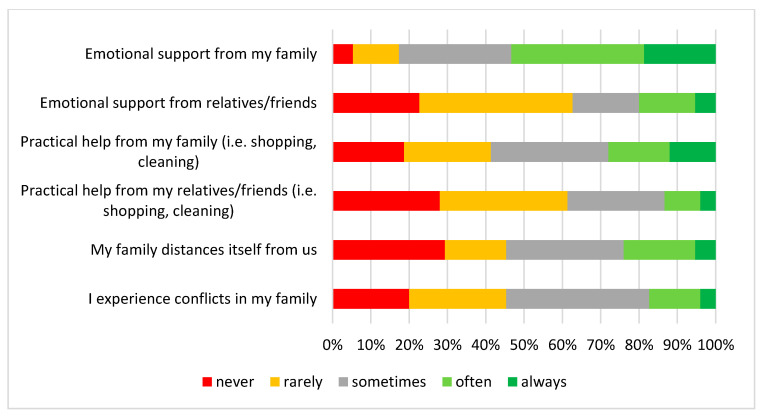
The impact of Dravet syndrome on family dynamics.

While over 54% of caregivers reported feeling supported by their family members, distant relatives and friends were indicated much less often (20%) (Figure 1). At the same time, many caregivers reported receiving little practical help in daily activities, such as shopping or cleaning either from their family (28%) or relatives and friends (13.4%).

**Figure 2:**
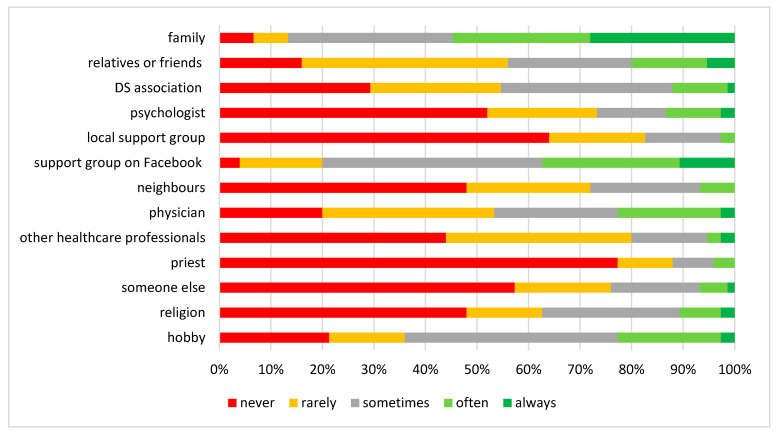
Coping resource of DS caregivers.

While the majority of caregivers declared that family was the main source of social support (54.6%), the internet and support groups on Facebook were also indicated as important sources of support (37.4%) (Figure 2). Additionally, 22.7% of caregivers pointed to physicians.

While in 18.6% of cases the diagnostic process lasted for less than a year, the majority of caregivers reported that it took up to three years (54.6%) (Table 3). A total of 5.4% of caregivers also declared that they had to struggle for accurate diagnosis for over 10 years. Only two caregivers (2.7%) reported that a correct diagnosis was made by the first physician who saw their DS child. On the other hand, almost half of caregivers reported that the diagnosis required visits to more than four physicians (49.3%), and in five cases more than 10 physicians (6.7%). For most caregivers, the basic source of knowledge about their child’s disease was the internet (88%) and medical specialists (57.3%).

While caregivers were dissatisfied with most aspects of healthcare services for DS children, the main causes of dissatisfaction were physicians’ knowledge about DS (77.3%), access to financial help with rehabilitation (75.5%) and government and social support for caregivers (68%) (Table 4). The quality of their medical care (57.3%) and physicians’ communication skills (57%) were rated relatively highly, as was access to medications for DS children (54.7%).

Neither financial situation nor domicile had an impact on the time of diagnosis (Table 5). The impact of the time taken for a diagnosis of a child in the caregiver’s opinion regarding the quality of the care system was clear. A relationship was found between the declared financial situation and the medical costs incurred as a result of the child’s disease, which may suggest that some caregivers have a problem in this regard.

## 4. Discussion

A growing body of literature reports that caregivers of DS children are under constant stress over the diagnosis and persistent severe seizures [11,14,20]. Medical co-morbidities, cognitive dysfunction, motor, behavioural and communication impairments, together with eating problems [9,13] also result in fear, anxiety, uncertainty and sleep problems [22,25,33], which seriously harm parents’ quality of life [27,28,29]. This should not come as a surprise since caregivers of children with other severe drug-resistant neurological disorders, including autism [34,35], Huntington disease [36,37], fragile X syndrome, Prader–Willi syndrome, Williams syndrome and 22q11.2 deletion syndrome [38,39,40,41,42], and other rare diseases [43,44,45] also report that numerous health problems, emotional lability and behavioural changes in their children seriously affect caregivers’ physical, mental and emotional health, family, social and economic life, result in social isolation, care overload and feeling burdened. At the same time, while caring for children with such neurological diseases can also be frightening and challenging, this research supports observations made by others that due to the complexity and severity of seizures in DS, which can lead to the deterioration of a child’s physical and cognitive health or premature death, caregiving for a DS child is far more challenging than other resistant epi-syndromes [29,46,47,48].

Consistent with previous findings, Polish DS caregivers enrolled in this study reported being burdened by their children’s health problems, mood swings, reduced mobility, behavioral changes and changes in personality, which influence many aspects of a caregivers’ daily life. As most parents declared themselves to be closely involved in the care for their DS children, they stressed a deterioration of their physical and mental health, fatigue, insomnia, eating problems and substance abuse. Similarly, caregivers enrolled in a multinational survey reported that caring for a DS child negatively affects caregivers’ everyday activities (91%), social interactions (80%) and family dynamics (70%) [24]. In the United States, parents of DS children reported that, while due to caregiving some tasks required more time—providing transportation (93%), patient’s personal care (87%), additional household tasks (83%), communication (80%) and observation of symptoms (77%)–others were perceived as more difficult, such as the arrangement of care (73%), communication (70%), co-ordination of resources (67%), management of patient behavior (67%) and personal care (63%) [11,22]. However, parents of children with autism, Willimas syndrome, 22q11.2 deletion syndrome and other rare diseases also report that key challenges related to the caregiving burden were the child’s behaviour, mood swings and psychiatric conditions [38,39,40,41,45].

This study also shows that although most caregivers felt supported by their families, they also reported the negative impact of carving for a DS child on their relationships with partners, other family members and friends. Although the vast majority of respondents expressed the need for respite from care and time for themselves, the majority felt alone, as both extended family members and friends provided little support. However, an earlier study on the emotional experiences of Polish DS carers showed that while 73.4% of parents felt overwhelmed by the caregiving role and had no time for their personal development, almost half complained about the negative impact of DS on their relationships with friends, and general well-being [33]. Also, Nolan et al. demonstrated that DS harmed caregivers’ family interactions (38%), interpersonal relations with friends (63%) and partners (54%) [46,47]. While Jensen showed that DS caregivers increasingly feel they are not understood by their families [11], Desnous et al. reported that 89% of DS parents declared that their children’s fever and seizures damaged their social interactions, and for 84% they had detrimental effect on their professional life [48]. Thus, although many caregivers of children with other neuro-developmental disorders also report that caregiving seriously affects their relationships with spouse/partner, healthy children and members of the extended family, it was suggested that in case of DS children these proportions are similar or even higher [41,49,50].

Many Polish caregivers also complained of having little time to themselves. Similarly, while 51% of Spanish caregivers reported having had less than one hour per week for themselves, 28% declared it was less than one hour per day, and 33% admitted not being able to go on holiday [27]. This problem was also highlighted in a multinational survey, in which 77% of DS carers reported having less than one hour per day to themselves [24].

Even though this study did not measure the direct and indirect financial costs resulting from caregiving responsibilities, it shows that in order to provide care for DS children, many parents face problems at work, have to quit their jobs or look for other work, while others are burdened economically due to missed days or the reduced number of working hours. This supports previous findings, which reported that, apart from many direct costs related to the necessity for substantial healthcare in the form of medication, emergency admissions, ambulance calls, epilepsy specialist visits, physiotherapy, parents face high indirect expenditures related to workdays lost, loss of salary or job [17,23,24,26,51,52]. A European study reported, for instance, that 27% of caregivers declared having lost their jobs, and 31% of those who worked had missed some working days due to their child’s DS. As many as 32% of Spanish caregivers similarly reported losing their jobs, 78% missed days from work or had a reduced salary, and 79% admitted that DS had affected their professional career [27]. Some 27% of American caregivers had to quit their jobs or take early retirement, 18% had to find alternative work and 18% lost their jobs. Nearly 64% also reported a salary change [23]. Similar results were reported in Germany [26].

Another important finding is that DS caregivers reported unsatisfactory experiences with the Polish healthcare system. Even though many respondents rated the quality of medical care for DS children highly, access to medication and doctors’ communication skills, they criticized access to information about DS, access to specialists and financial support for rehabilitation. They also complained about doctors’ knowledge about DS and practical information about the disease. Respondents were also dissatisfied with the support they received from healthcare professionals and the lack of empathy among doctors. Thus, this study confirms observations made by others that healthcare professionals lack of knowledge on rare neurological disorders is one of the main barriers of access to the healthcare system [37,39,49,53,54].

Previous studies have likewise shown that DS caregivers experience a stressful relationship with the healthcare system and complain that their specific health concerns were ignored by healthcare professionals who often take no account of parents’ points of view [11,47]. Many also feel that their physical, mental and social health is often overlooked in clinical research [11,29]. Similarly, caregivers of children with such neurological disorders as autism, 22q11DS, Willimas syndrome or Huntington disease complain of insufficient empathy from healthcare professionals and feel forgotten by the healthcare system [34,35,43,44,45,49,55,56].

Many also complained at the so-called diagnostic odyssey typical of rare diseases [33,57,58,59]. This is crucial because late diagnosis or misdiagnosis often results in delayed access to appropriate treatment, drugs and specialized rehabilitation programs [60]. This study therefore confirms that DS parents experience problems in receiving a timely, accurate diagnosis. A total of 80% of Spanish DS caregivers also reported delayed diagnoses or misdiagnoses [27]. Another study reported that for 50% of DS caregivers the diagnostic journey took up to three years after the initial seizure. For 23% it was more than 5 years and for 8% more than 10 years. Some 68% of caregivers consulted more than three specialists before the final DS diagnosis was received, and 29% consulted five or more neurologists [17]. Our findings suggest that during the diagnostic process, parents of DS children often struggle to obtain accurate information and appropriate care. This, however, should come as no surprise, as previous Polish studies have demonstrated a lack of knowledge and experience from healthcare practitioners, including doctors and nurses regarding rare disease [61,62,63]. At the same time, in some cases caregivers of children with other neurological disorders report even more problems with the diagnostic and therapeutic odyssey and the ignorance of doctors and other health professionals [55,56,64,65].

All in all, this study supports previous findings which show that although most DS carers are primarily challenged by the physical, mental and behavioural state of their DS children, they also lack coping resources, and do not feel supported by their families, government and social institutions and healthcare professionals. Consequently, most caregivers are under constant stress and experience helplessness, depression, anxiety, fear, physical fatigue, mental exhaustion and financial burden [33]. Thus, while parents of children with other neurological disorders, i.e., autism, fragile X syndrome, Prader–Willi syndrome, Williams syndrome or 22q11.2 deletion syndrome also feel burdened by their child’s conditions, mood swings and behaviours, and experience high levels of emotional distress, anxiety and depression [34,35,36,37,38,39,40,41,42], they also report higher life satisfaction and better interactions with their families, while in DS such negative emotions are at similar or higher level [20,21,28,33]. 

This study has its limitations, which should be considered when interpreting the findings. Firstly, as only 75 caregivers completed the questionnaire, these findings do not allow generalizations in context to a larger population of Polish DS carers. However, because Poland still lacks a single registry of DS children, it was not possible to access the entire population of DS patients and their caregivers in the country. Secondly, this study focused on paediatric patients and may not reflect opinions of parents who provide care for older persons suffering from DS. Thirdly, as this study was focused of caregivers’ experiences it asked no questions about DS children’s clinical condition or symptoms that influence caregivers’ experiences and feeling of being burdened. Fourthly, this study may be biased due to the web-based request for participation and the on-line format of the survey. The results may consequently fail to reflect the opinions of those caregivers who are not affiliated with the Polish support group of the DRAVET.PL association, do not use its social media and feel uncomfortable speaking personally about their experiences. Fifthly, because the data were collected using an original questionnaire comprising predefined questions, it made spontaneous reporting from caregivers impossible. Some important challenges and needs faced by the caregiver may therefore not have been captured. Although there are several tools for assessing the burden of caregiving for a number of conditions, none were developed to assess problems and needs of DS caregivers. Moreover, the instrument used in this study was an ad hoc tool and was not validated. Consequently, there is a risk of measurement error. Sixthly, this study’s primary focus of interest may have led to an over-representation of unsatisfactory experiences. Finally, the vast majority of respondents were female (F:M 68:7), mainly mothers, so the results may not reflect the experiences of fathers or other male caregivers, whose experiences may be different [66,67]. This gender gap in caregiving was, however, also reported in previous studies that reported that it is frequently the mother who takes on the role of the main caregiver [11,24,28]. 

There are also some advantages of this study. Most importantly, as, to our knowledge, this is the first study on the problems and needs of caregivers of DS children in Poland, it sheds a new light on the experiences on this important topic. Additionally, as it gave voice to DS caregivers and enabled them to present their perspective it has helped to identify challenges related to caring for DS children and may stimulate further research on factors affecting the experiences and needs of Polish DS caregivers.

## 5. Conclusions

While healthcare professionals focus on DS patients, their caregivers’ problems and needs are often overlooked. DS carers can therefore be described as ‘the forgotten ones’ in terms of the Dravet syndrome. Meanwhile, the impact of caring for DS children goes far beyond clinical facets and seriously affects every aspect of caregivers’ lives, including their health, quality and satisfaction of life, everyday activities, family and professional life, social interactions, and may be a source of financial burden. Thus, in order to enhance caregivers’ well-being, efforts should therefore focus on developing a holistic approach that should include the entire DS family.

## Figures and Tables

**Table 1:** Demographic characteristics of DS caregivers involved in the study.

	N (%)
*Relationship with DS child*	
mother	66 (88)
father	7 (9.3)
other relative (grandmother, sister)	2 (2.7)
*Caregiver’s age*	
>30	2 (2.7)
30–39	32 (42.6)
40–49	35 (46.7)
<50	6 (8)
*Number of children in the family diagnosed with DS*	
1	71 (94.7)
2 or more	4 (5.3)
*Child’s sex*	
girl	37 (46.2)
boy	43 (53.8)
*Child’s age (in years)*	
under 1	2 (2.5)
2–3	3 (3.7)
4–5	10 (12.5)
6–10	28 (35)
11–18	35 (43.8)
missing	2 (2.5)
*Extracurricular help for your DS child (* *hours per week)*	
1–6	7 (9.4)
7–15	4 (5.3)
< 16	4 (5.3)
I do not use any extra help	60 (80)
*Care allowance*	
yes	67 (89.3)
no	8 (10.7)
*Place of residence*	
up to 10,000 inhabitants	24 (32)
10–50,000 inhabitants	13 (17.3)
51–100,000 inhabitants	8 (10.7)
101–500,000 inhabitants	16 (21.3)
above 500,000 inhabitants	14 (18.7)
*Severity of DS child’s health condition and disability*	
very severe	51 (68)
severe	14 (18.7)
moderate	9 (12)
mild	1 (1.3)
none	0 (0)
*The impact of DS on family*	
DS has not affected my relationship	36 (48)
DS has affected my relationship but not resulted in breakup	33 (44)
DS has strengthened my relationship with the second parent	1 (1.3)
relationship ended after DS diagnosis	2 (2.7)
relationship ended as caregiving for DS child became more demanding	1 (1.3)
other answer	2 (2.7)

**Table 2:** Challenges related to caregiving over a DS child.

	Never	Rarely	Sometimes	Often	Always
*What makes caring for DS child challenging*					
reduced mobility	6 (8)	12 (16)	14 (18.7)	26 (34.6)	17 (22.7)
personality changes	7 (9.3)	9 (12)	22 (29.3)	23 (30.7)	14 (18.7)
mood swings	5 (6.7)	10 (13.3)	17 (22.7)	27 (36)	16 (21.3)
changes in behaviour	4 (5.3)	10 (13.3)	22 (29.4)	19 (25.3)	20 (26.7)
communication problems	4 (5.3)	10 (13.3)	27 (36)	21 (28)	13 (17.4)
drug and healthcare expenses	6 (8)	10 (13.3)	21 (28)	23 (30.7)	15 (20)
expenses related to adapting the home to the child’s needs	16 (21.3)	19 (25.3)	23 (30.7)	11 (14.7)	6(8)
lack of access specialised care equipment	18 (24)	20 (26.7)	17 (22.7)	11 (14.7)	9 (12)
lack of access to medications	12 (16)	26 (34.7)	20 (26.7)	10 (13.3)	7 (9.3)
problems with drug reimbursement or purchase of drugs	10 (13.3)	24 (32)	21 (28)	13 (17.3)	7 (9.3)
lack of access to rehabilitation	3 (4)	11 (14.7)	19 (25.3)	28 (37.3)	14 (18.7)
*Which of the aspects of daily life are burdensome?*					
maintenance of the house	5 (6.7)	14 (18.6)	26 (34.7)	24 32)	6 (8)
financial issues	5 (6.7)	15 (20)	29 (38.7)	17 (22.7)	9 (12)
transport	15 (20)	18 (24)	20 (26.7)	20 (26.6)	2 (2.7)
caregiving for DS child	3 (4)	10 (13.3)	18 (24)	29 (38.7)	15 (20)
caring for a healthy child	29 (38.7)	16 (21.3)	16 (21.3)	12 (16)	2 (2.7)
lack of time for myself	1(1.3)	2 (2.7)	15 (20)	36 (48)	21 (28)
problems at work due to caregiving responsibilities	22(29.3)	9 (12)	6 (8)	14 (18.7)	24 (32)
work restrictions due to caregiving responsibilities	8(10.7)	6 (8)	7 (9.3)	4 (5.3)	50 (66.7)
*Do you experience any of these problems?*					
eating problems/lack of appetite	14(18.7)	32 (42.6)	19 (25.3)	8 (10.7)	2 (2.7)
weight loss/ gain weight	11(14.7)	16 (21.3)	22 (29.3)	23 (30.7)	3 (4)
fatigue	1(1.3)	2 (2.7)	9 (12)	40 (53.3)	23(30.7)
problems with sleeping/insomnia	7(9.3)	11 (14.7)	25 (33.3)	23 (30.7)	9 (12)
deterioration of physical health	7(9.3)	6 (8)	22 (29.4)	34 (45.3)	6 (8)
deterioration of mental health	5(6.7)	4 (5.3)	21 (28)	34 (45.3)	11 (14.7)
intimacy problems with spouse/partner	5(6.7)	13 (17.3)	17 (22.7)	29 (38.7)	11 (14.7)
substance abuse (cigarettes, alcohol, medications)	32(42.7)	19 (25.3)	12 (16)	11 (14.7)	1 (1.3)

**Table 3:** Diagnostic odyssey in Dravet syndrome.

	N (%)
*Time spent waiting for a diagnosis? (in years)*	
>1	14 (18.6)
1–2	27 (36)
2–3	14 (18.6)
4–5	8 (10.7)
6–9	8 (10.7)
<10	4 (5.4)
*Number of physicians consulted before DS diagnosis was received*	
1	2 (2.7)
2–3	31 (41.3)
4–6	28 (37.3)
7–10	9 (12)
More than 10	5 (6.7)
*Source of information on DS*	
internet	66 (88)
medical specialist	43 (57.3)
family doctor	6 (8)
local support group	17 (22.7)
genetic clinic	6 (8)
scientific publications	28 (37.3)
association/foundation for people with DS	39 (52)
other (Facebook, friends with DS children)	6 (8)

**Table 4:** DS caregivers’ perception of healthcare services.

	Very Bad	Rather Bad	I Do Not Know	Rather Good	Very Good
Support for caregivers from government and social institutions	19 (25.3)	32 (42.7)	11 (14.7)	13 (17.3)	0 (0)
Quality of health service for your DS child	5 (6.7)	21 (28)	6 (8)	37 (49.3)	6 (8)
Availability of specialist consultations (neurologist, geneticist, psychologist)	17 (22.7)	26 (34.7)	6 (8)	24 (32)	2 (6.7)
Access to medications for DS children	14 (18.6)	17 (22.7)	3 (4)	41 (54.7)	0 (0)
Access to financial help with rehabilitation for DS children	21 (28)	35 (47.7)	6 (8)	13 (17.3)	0 (0)
Access to information on DS	19 (25.4)	25 (33.3)	7 (9.3)	23 (30.7)	1 (1.3)
Support for DS children and caregivers from healthcare professionals	12 (16)	38 (50.7)	10 (13.3)	12 (16)	3 (4)
Physicians’ knowledge about DS	17 (22.7)	41 (54.6)	6 (8)	11 (14.7)	0 (0)
Physicians’ practical information about DS (how to provide care for your DS child; how to perform various tasks)	12 (16)	33 (44)	10 (13.3)	18 (24)	2 (2.7)
Physician’s/neurologist’s/geneticist’s communication skills	10 (13.3)	16 (21.3)	7 (9.3)	36 (48)	6 (8)
Support caregivers receive from physicians	13 (17.3)	34 (45.4)	12 (16)	15 (20)	1 (1.3)
Physicians’ empathy	7 (9.3)	29 (38.7)	11 (14.6)	26 (34.7)	2 (2.7)
Contact with genetic clinic	6 (8)	13 (17.3)	29 (38.7)	25 (33.3)	2 (2.7)
Contact with psychological clinic	10 (13.3)	11 (14.7)	29 (38.7)	24 (32)	1 (1.3)

**Table 5:** Kendall’s rank correlation analysis.

	Tau B	95%CI	*p*
Child’s age: perceived health problems of child	0.082	−0.118; 0.269	ns
Time of diagnosis: domicile	0.038	−0.153; 0.229	ns
Time of diagnosis: perception of healthcare services	−0.313	−0.471; −0.145	<0.001
Time of diagnosis: financial situation	−0.107	−0.296; 0.088	ns
Medical expenses: contact with the healthcare system	0.261	0.085; 0.427	<0.01
Perception of healthcare services: domicile	−0.051	−0.220; 0.126	ns
Perception of healthcare services: financial situation	0.081	−0.122; 0.294	ns
Medical expenses: financial situation	−0.423	−0.593; −0.222	<0.001
Perceived health problems of a child: perception of healthcare services	−0.127	−0.298; 0.054	ns

ns: not significant

## Data Availability

The datasets generated during the study are available from the corresponding author on reasonable request.
